# Risk factors for length of NICU stay of newborns: A systematic review

**DOI:** 10.3389/fped.2023.1121406

**Published:** 2023-03-13

**Authors:** Maoling Fu, Wenshuai Song, Genzhen Yu, Yaqi Yu, Qiaoyue Yang

**Affiliations:** ^1^ Department of Nursing, Tongji Hospital, Tongji Medical College, Huazhong University of Science and Technology, Wuhan, China; ^2^ School of Nursing, Tongji Medical College, Huazhong University of Science and Technology, Wuhan, China

**Keywords:** length of stay, NICU, newborns, risk factors, systematic review

## Abstract

**Background:**

The improvement in survival of preterm infants is accompanied by an increase in neonatal intensive care unit (NICU) admissions. Prolonged length of stay in the NICU (LOS-NICU) increases the incidence of neonatal complications and even mortality and places a significant economic burden on families and strain on healthcare systems. This review aims to identify risk factors influencing LOS-NICU of newborns and to provide a basis for interventions to shorten LOS-NICU and avoid prolonged LOS-NICU.

**Methods:**

A systematic literature search was conducted in PubMed, Web of Science, Embase, and Cochrane library for studies that were published in English from January 1994 to October 2022. The PRISMA guidelines were followed in all phases of this systematic review. The Quality in Prognostic Studies (QUIPS) tool was used to assess methodological quality.

**Results:**

Twenty-three studies were included, 5 of which were of high quality and 18 of moderate quality, with no low-quality literature. The studies reported 58 possible risk factors in six broad categories (inherent factors; antenatal treatment and maternal factors; diseases and adverse conditions of the newborn; treatment of the newborn; clinical scores and laboratory indicators; organizational factors).

**Conclusions:**

We identified several of the most critical risk factors affecting LOS-NICU, including birth weight, gestational age, sepsis, necrotizing enterocolitis, bronchopulmonary dysplasia, and retinopathy of prematurity. As only a few high-quality studies are available at present, well-designed and more extensive prospective studies investigating the risk factors affecting LOS-NICU are still needed in the future.

## 1. Introduction

With advancements in perinatal medicine and the development of neonatal intensive care units (NICUs), neonatal mortality has declined in most parts of the world (1–3). The survival rates of extremely preterm infants have also increased from 76.0% in 2012 to 78.3% in 2018 (4–6). At the same time, the number of newborns requiring intensive care has increased, with NICU admissions rising from 6.4% in 2007 to 7.2% in 2018 (7, 8); therefore, prolonged length of stay (LOS) of these high-risk newborns admitted to the NICU has become a concern.

Prolonged LOS-NICU causes adverse effects on the newborn, the family, the medical staff, and even the hospital. For newborns, prolonged LOS-NICU exposes them longer to the hospital environments, including the noise, bright light, hospital-acquired infections, and so on, which would lead to a higher incidence of neonatal complications (9, 10). Some studies found that these exposures could affect the future development of newborns, such as developmental retardation, predisposition to chronic illness, impairment of cognitive function, and neurodevelopmental disorders (11–13). Prolonged LOS-NICU could also prevent the establishment of parent–newborn interactions and increase the cost of hospitalization (14, 15), which may cause severe emotional and financial stress to families (16). Moreover, parents are concerned about how long their newborn needs to stay in the NICU. Accurate information about the LOS may alleviate unnecessary anxiety for parents. However, it is often difficult for medical staff to make accurate predictions when parents consult with them. From the healthcare system perspective, a prolonged LOS-NICU could reduce the utilization rate of beds and exacerbate the problem of inadequate healthcare resources (17). To reduce unnecessary LOS and avoid prolonged LOS-NICU, we must determine the risk factors affecting the LOS-NICU of newborns.

Determining the risk factors for the LOS-NICU of newborns is conducive to improve the ability of predicting LOS-NICU accurately, which is critical for planning hospital resources, counseling families, stimulating quality improvement initiatives, and effectively avoiding prolonged LOS-NICU of newborns (18, 19). However, there is little evidence related to this issue. Moreover, most of the available evidence comes from observational studies, which may lead to selection bias. In addition, some important risk factors identified in single-center studies might be found only by chance because of the level of medical institutions [there are currently four categories of NICUs (20): Level I NICU: well newborn nursery, which offers regular nursery care for healthy newborns; Level II NICU: special care nursery, which cares for premature and sick newborns; Level III NICU: neonatal intensive care unit, which cares for seriously ill newborns; Level IV NICU: regional neonatal intensive care unit, which cares for the most critically ill newborns and babies), which might not apply to other hospitals. Therefore, the purpose of this review was to determine the risk factors for the LOS-NICU of newborns from multiple studies and to evaluate the evidence that currently exists.

## 2. Methods

The review followed the PRISMA reporting guidelines, a 27-item list designed to improve the reporting of systematic evaluations (21), and was registered with PROSPERO (registration number: CRD42022370357). All relevant analyses were based on previously published studies and did not require ethical approval or patient consent.

### 2.1. Search strategy

A systematic literature search was conducted in PubMed, Web of Science, Embase, and Cochrane library for studies that were published in English from January 1994 to October 2022 using keywords, Medical Subject Headings (MeSH), and other index terms, as well as combinations of these terms and appropriate synonyms. The search terms focused on “Infant, Newborn”, Infants, “Intensive Care Units, Neonatal”, “NICU”, “Length of stay”, “Stay Length”, “Risk factors”, “Influencing factors”, Predictors, and their synonyms (see the Supplementary Material for the complete search strategy). In addition, the reference lists of all selected studies were manually searched for any additional studies that met the criteria.

### 2.2. Inclusion criteria

• Newborns, preterm infants, or low-birth-weight infants admitted to the NICU were used as the study population. Low-birth-weight infants (LBWI) are defined as infants with a birth weight of less than 2,500 g, regardless of gestational age or maturity.
• LOS-NICU as the primary outcome indicator and risk factors for LOS-NICU as the primary study objective.
• Prospective or retrospective cohort study.
• Multivariate data analysis using more than two covariates, which could be from multivariate models (e.g., logistic regression models, multiple linear regression models, and Cox regression models).
• With the use of conventional surfactants and the introduction of prenatal steroids, neonatal survival rates significantly improved in the year 1994 compared with the previous years (22), so this review was retrieved from that year.
• Studies published in English.

### 2.3. Exclusion criteria

• Wrong study population, such as newborns in general pediatrics or pregnant and lying-in woman.
• Wrong study outcomes include death, hospitalization costs, and readmission to the NICU.
• A specific disease area (e.g., congenital heart disease), because these newborns may differ significantly from other newborns in the NICU.
• Conference proceedings, review articles, letters, and editorials.
• Single-factor analysis for an influential factor, without controlling for confounding factors.
• Clinical trial articles, because the clinical trial population cannot replace other NICU newborns.
• The original article could not be found in various ways.
• They were not published in English.

### 2.4. Data extraction

Two reviewers extracted data on the characteristics and outcomes of studies using a Microsoft Excel 2019 spreadsheet. The extraction process was performed independently, in duplicate, and with a third senior reviewer resolving disagreements when necessary. For each included study, data were collected on the following characteristics: (1) basic information about the study, including first author, country, year of publication, study duration, study design, and type of data analysis; and (2) basic and essential information of the study population, including exclusion criteria, sample size, and major risk factors.

### 2.5. Quality assessment

The risk of bias for each eligible study was assessed using the Quality in Prognostic Studies (QUIPS) tool recommended by the Cochrane Prognostic Methods Group (23). Since the purpose of prognostic studies is to predict a specific outcome on the basis of a range of possible factors, prognostic factors are the focus of prognostic studies and confounding factors are not considered relevant to predict outcomes. Therefore, a modified version of the QUIPS tool was used to discuss the quality of the study. This quality assessment method considers five domains of potential biases: (1) study population; (2) study attribution; (3) risk factor measurement; (4) outcome measurement; and (5) statistical analysis and reporting. The assessment of the study population contained five questions with a maximum score of 3 for each question, and the other four domains each contained three questions with a maximum score of 5 for each question.

We used the predesigned risk of bias assessment form to score a range of questions within each of the five domains on the basis of the adequacy of reporting, with a maximum total score of 75. If the total score was ≥60, the study was considered to have a low overall risk of bias and was classified as a “high-quality” study; if the total score was 45–59, the study was considered to have a medium risk of bias and was classified as a “medium-quality” study; if the total score was <45, the study was considered to have a high overall risk of bias and was classified as a “low-quality” study. In order to ensure the accuracy of assessment, a third reviewer extracted data from five randomly selected studies and reviewed these studies for risk of bias and methodological quality.

## 3. Results

**Figure 1:**
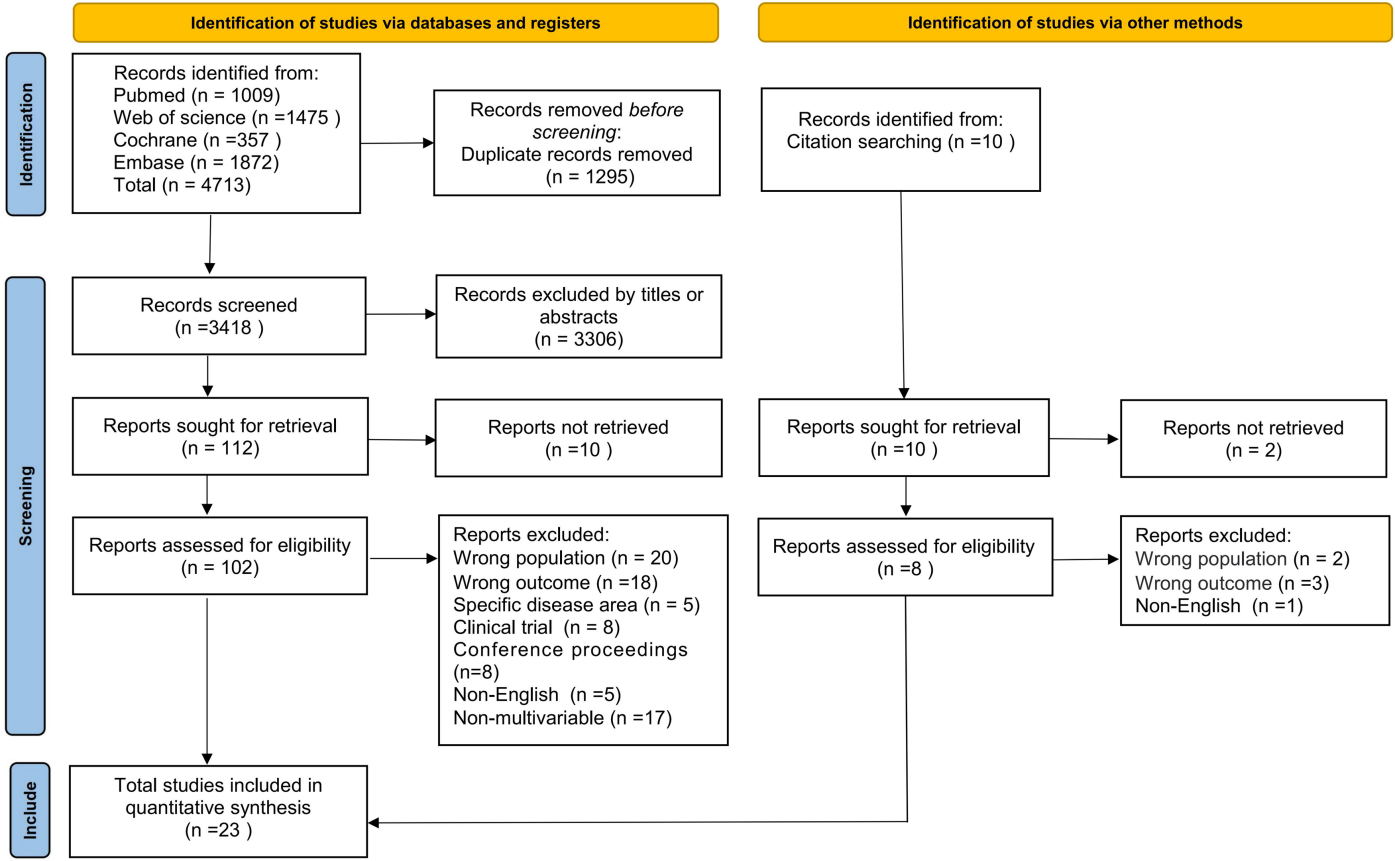
Flow chart of the systematic literature search.

The literature search of the databases identified 4,713 potentially relevant articles, and 1,295 duplicates were excluded, leaving 3,418 articles to be screened for title and abstract. After the eligibility assessment, the full text of 102 articles was retrieved, of which 21 met the inclusion criteria. We searched the list of all the references included in the study and conducted a full text search of 10 articles, among which 2 original texts could not be found. According to the strict inclusion and exclusion criteria, two articles were determined to meet the requirements. Ultimately, 23 articles were identified for inclusion in this review. Study identification is summarized in Figure 1.

Of these studies, four were prospective studies (24–27) and the remaining 19 studies were retrospective (1, 9, 18, 28–43), of which 14 were multicenter studies (9, 24, 25, 27–29, 31, 33–35, 37–39, 42) and 9 were single-center studies (1, 18, 26, 30, 32, 36, 40, 41, 43). The sample size ranged from 191 to 7,60,037. Two studies with the largest sample sizes included 23,551 newborns (34) and 7,60,037 newborns (38). Six of these studies constructed clinical prediction models (9, 18, 27, 34, 42, 43). The basic characteristics of the included literature are given in Table 1.

**Table 1:** Summary characteristics of the 23 studies included in this review.

Author	Country of study	Year of publication (data)	Study design	Type of analysis	Exclusions in study	Study population	Number of patients	Statistical methods	Study quality
Bender et al. (18)	America	2013 (1999 and 2002)	Retrospective	Multivariable (prediction model)	(1) Died prior to NICU; (2) admission for preterminal comfort care; (3) congenital anomaly	All gestations	908	Log-logistic model and Weibull model	Moderate
Berry et al. (28)	Canada	2008 (2002)	Retrospective	Multivariable	Admissions for convalescent or step-down care	All gestations	625	Logistic regression	Moderate
Charlton et al. (35)	America	2019 (2014)	Retrospective	Multivariable	(1) No IVFs for at least 48 h; (2) age >7 days at NICU admission; (3) congenital heart disease with surgery at <7 days of life; (4) congenital anomaly; (5) NICU deaths; (6) severe kidney anomaly	Newborns with intravenous fluids longer than 48 h	2,110	Linear regression	Moderate
Ge et al. (1)	China	2022 (2016–2021)	Retrospective	Multivariable	(1) congenital anomaly; (2) transfer; (3) NICU deaths; (4) discharge against medical advice	28–31 weeks gestational age	709	Two-piece-wise linear regression model	High
Hintz et al. (9)	America	2010 (2002–2005)	Retrospective	Multivariable (prediction model)	(1) chromosomal or congenital anomalies; (2) In hospital one year; (3) transfer; (4) missing data; (5) NICU deaths	<27 weeks gestational age	2,254	Linear and logistic regression models	High
Jetton et al. (29)	America	2017 (2014)	Retrospective	Multivariable	(1) No IVFs for at least 48 h; (2) age >14 days at NICU admission; (3) congenital heart disease with surgery at <7 days of life; (4) lethal chromosomal anomaly; (5) mortality <48 h; (6) severe, bilateral congenital renal disease	Newborns with intravenous fluids longer than 48 h	2,022	Linear regression	High
Kheiry et al. (43)	Iran	2020 (2016–2018)	Retrospective	Multivariable (prediction model)	Not mentioned	All gestations	603	Cox model and four parametric models	Moderate
Klinger et al. (37)	Israel	2005 (1995–2002)	Retrospective	Multivariable	NICU deaths	<1,500 g birth weight	9,310	Logistic regression	High
Lee et al. (27)	America	2013 (2008–2010)	Prospective	Multivariable (prediction model)	(1) NICU deaths; (2) transfer; (3) congenital anomaly; (4) surgeries	401–1,000 g birth weight	2,012	Linear mixed model	High
Lee et al. (34)	America	2016 (2008–2011)	Retrospective	Multivariable (prediction model)	(1) congenital anomaly; (2) readmission; (3) NICU deaths; (4) surgeries; (5) weight gain of 65 g per day	401–1,500 g birth weight; 22–29 weeks gestational age; other larger and older infants	23,551	Negative binomial Generalized linear model	Moderate
Eken et al. (30)	Turkey	2016 (2012–2014)	Retrospective	Multivariable	(1) NICU deaths; (2) transfer; (3) missing data	All gestations	3,607	Linear regression	Moderate
Eken et al. (36)	Turkey	2017 (2013–2015)	Retrospective	Multivariable	(1) Missing data; (2) transfer; (3) twin pregnancies; (4) congenital anomaly	24–34 weeks of gestation following PPROM	331	Linear regression	Moderate
Murki et al. (24)	India	2020 (2016–2018)	Prospective	Multivariable	(1) Age >1 days at NICU admission; (2) readmission; (3) NICU deaths; (4) transfer	25–33 weeks gestational age	3,095	Linear regression	Moderate
Patil et al. (38)	America	2021 (1997–2018)	Retrospective	Multivariable	(1) Congenital anomaly; (2) chromosomal abnormalities/aneuploidies	22–36 weeks gestational age	760,037	Logistic regression	Moderate
Payne et al. (39)	America	2004 (1998–1999)	Retrospective	Multivariable	(1) NICU deaths; (2) transfer; (3) early-onset sepsis; (4) congenital anomaly; (5) missing data	<1,500 g birth Weight	2,809	Logistic regression	Moderate
Pepler et al. (42)	South Africa	2012 (2007–2008)	Retrospective	Multivariable (prediction model)	(1) NICU deaths; (2) transfer; (3) missing data	(1) 401–1,500 g birth weight; (2) 22–29 weeks gestational age (3) Age <28 days at NICU admission	3,703	Log-linear models	Moderate
Różańska et al. (25)	Poland	2015 (2009–2013)	Prospective	Multivariable	(1) Missing data; (2) NICU deaths	<1,500 g birth weight	2,003	Generalized linear model	Moderate
Sahiledengle et al. (26)	Ethiopia	2020 (2018–2019)	Prospective	Multivariable	(1) Died prior to NICU; (2) congenital anomaly	All gestations	191	Cox regression	Moderate
Shah et al. (40)	Eritrea	2012 (2006)	Retrospective	Multivariable	NICU deaths	All gestations	953	Linear regression	Moderate
Singh et al. (31)	India	2021 (2018–2019)	Retrospective	Multivariable (prediction model)	(1) Congenital anomaly; (2) palliative care; (3) request for discharge; (4) transfer; (5) NICU deaths	All gestations	1,047	Log model	Moderate
Xie et al. (32)	China	2022 (2012–2020)	Retrospective	Multivariable	(1) Missing data; (2) readmission; (3) hospitalization for <1 day	All gestations	16,094	Cox regression	Moderate
Ye et al. (41)	China	2011 (2006–2009)	Retrospective	Multivariable	(1) NICU deaths; (2) transfer	24–34 weeks of gestation following PPROM	289	Linear regression	Moderate
Zhang et al. (33)	China	2022 (2019)	Retrospective	Multivariable	(1) Congenital anomaly; (2) missing data; (3) NICU deaths; (4) discharge against medical advice; (5) transfer to nonparticipating hospitals	<32 weeks gestational age	6,580	Linear regression	Moderate

NICU, neonatal intensive care unit.

### 3.1. Study populations within LOS-NICU studies

The studies investigated a range of newborns with different gestational ages and different birth weights, of which seven studies were conducted with preterm infants as respondents (1, 9, 24, 33, 36, 38, 41); four studies were conducted in low birth weight infants as respondents (25, 27, 37, 39); two studies included only newborns who were recipients of intravenous (IV) fluids for at least 48 h, based on the purpose of the study, in order to enroll infants most likely to have serial serum creatinine (SCR) and urine output (UOP) measurements (29, 35); two studies made different requirements for gestational age or birth weight (34, 42); and the other eight studies included all eligible newborns without other requirements for gestational age or birth weight (18, 26, 28, 30–32, 40, 43).

### 3.2. Exclusion criteria of LOS-NICU studies

Exclusion criteria mainly included the following: death during NICU stay or prior to admission (1, 9, 18, 24, 26, 27, 29–31, 33–35, 37, 39–42); lethal chromosomal abnormalities or severe congenital malformations (1, 9, 18, 26, 27, 29, 31, 33–36, 38, 39); missing information on LOS-NICU or other key information (25, 30, 32, 33, 36, 39); transfer to another NICU or long-term care facility (1, 9, 24, 27, 30, 31, 33, 36, 39, 41, 42); and discharge against medical advice (1, 31, 33). In addition, exclusions included surgery (27, 29, 34, 35); readmission after initial discharge (24, 32, 34); LOS-NICU less than 24 h (32) or more than 1 year (9); and admission 24 h (24), 7 days (35), or 14 days (29) after birth and admission for convalescent, step-down care or preterminal comfort care (18, 28, 31).

### 3.3. Quality of the LOS-NICU studies

Five of the included studies were of high quality (1, 9, 27, 29, 37), 18 were of moderate quality (18, 24–26, 28, 30–36, 38–43), and none were of low quality. There were few issues related to study participation, as in most studies, information was obtained from medical records and electronic databases, which would possibly introduce a low risk of bias. Study attrition due to newborns being transferred out of the hospital or study coverage area was a potential issue, with only one of the studies continuing follow-up of transferred newborns until they were discharged (33). With regard to the definition of LOS-NICU, four studies were classified. Among them, Berry et al. classified LOS-NICU as <21 days and ≥21 days (28), Klinger et al. classified it as non-delayed and delayed discharge (defined as discharge at a postmenstrual age (PMA) greater than 42 completed weeks) (37), Ge et al. and Hintz et al. classified discharge as early and late discharge (PMA at discharge was in the fourth quartile among newborns born at the same gestational age) (1, 9), and the remaining studies used continuous LOS-NICU/ PMA as the primary outcome indicator. All studies reported at least one apparent risk factor that could be measured objectively.

In general, study quality was considered good with a low level of potential bias. Because of significant heterogeneity in the study design, study population, type of outcome indicator, and method of statistical analysis, it was not feasible to conduct a meta-analysis.

### 3.4. Risk factors in LOS-NICU studies

The 23 included studies described 58 statistically significant risk factors for LOS-NICU identified by multivariable analysis. These variables are grouped into six broad categories: inherent factors (73.9%, 17/23); antenatal treatment and maternal factors (34.7%,8/23); diseases and adverse conditions of the newborn (78.3%,18/23); treatment of the newborn (21.7%,5/23); clinical scores and laboratory indicators (43.5%,10/23); and organizational factors (34.7%,8/23). Details of the risk factors identified in each study are given in Table 2.

**Table 2:** Risk factors for influencing LOS-NICU included in the analysis of each study.

Risk factors	Bender et al.	Berry et al.	Charlton et al.	Ge et al.	Hintz et al	Jetton et al.	Kheiry et al.	Klinger et al.	Lee et al.	Lee et al.	Eken et al	Eken et al	Murki et al.	Patil et al.	Payne et al.	Pepler et al.	Różańska et al.	Sahiledengle et al.	Shah et al.	Singh et al.	Xie et al.	Ye et al.	Zhang et al.	Number of studies
Inherent factors																								17
Birth weight	X				X			X	X	X	X	X	X			X	X	X	X		X	X		14
Gestational age	X										X		X				X	X	X	X	X	X	X	10
SGA								X	X	X			X						X				X	6
Congenital anomalies		X						X			X		X											4
Sex									X	X										X	X			4
Ethnicity/race/nationality									X	X						X								3
Year of birth										X														1
Multiple gestation										X														1
Discharge weight																						X		1
Antenatal treatment and maternal factors																								8
Mode of delivery										X										X	X	X	X	5
Maternal hypertension									X	X													X	3
Antenatal steroids									X	X														2
Abnormal antenatal umbilical artery Doppler													X											1
Primigravida																							X	1
Maternal diabetes										X														1
Maternal age										X														1
Received prenatal care										X														1
Other maternal conditions										X														1
Preeclampsia												X												1
Diseases and adverse conditions																								18
Sepsis					X			X			X		X							X		X	X	7
NEC					X			X				X	X										X	5
BPD					X			X				X	X										X	5
ROP					X						X	X											X	4
Neonatal AKI			X			X	X																	3
RDS											X		X							X				3
At least one infection (including HIAs, NBIs)															X		X	X						3
Fetal distress									X	X														2
CCD											X	X												2
IVH								X															X	2
Pneumonia											X								X					2
PDA											X	X												2
TTNB											X									X				2
IUGR											X													1
Congenital metabolic disorders											X													1
Seizures													X											1
NNH																				X				1
Comorbidities																					X			1
PVL																							X	1
Treatment of the newborn																								5
Surgery		X			X																			2
Resuscitation													X											1
Nutrition deviation																				X				1
Medication deviation																				X				1
Breastfeeding							X																	1
Phototherapy							X																	1
Mechanical ventilation							X																	1
CVC							X																	1
Clinical scores and laboratory indicators																								10
SNAPPE/SNAPPE-II score	X	X																						2
5-min Apgar score <4									X	X														2
CRIB score																	X							1
TRIPS score																							X	1
NT-proBNP7				X																				1
Hassan scale														X										1
Bacterial culture of cord blood																						X		1
1-min Apgar score																X								1
MAIN scores	X																							1
Organizational factors																								5
Center (random effect)					X				X															2
Transferred/outborn status										X											X			2
Obstetrical conditions										X														1
Children's hospitals vs. prenatal centers																							X	1

SGA, small for gestational age; NEC, necrotizing enterocolitis; BPD, bronchopulmonary dysplasia; ROP, retinopathy of prematurity; AKI, acute kidney injury; RDS, respiratory distress syndrome; HIAs, hospital-acquired infection; NBIs, nosocomial bloodstream infections; CCD, congenital cardiac disease; IVH, intraventricular hemorrhage; PDA, patent ductus arteriosus; TTNB, transient tachypnea of the newborn; IUGR, intrauterine growth retardation; NNH, neonatal hyperbilirubinemia; PVL, periventricular leukomalacia; CVC, central venous catheter; SNAPPE, Score for Neonatal Acute Physiology; CRIB, Critical Risk Index for Babies; TRIPS, Transport risk index of physiologic stability; MAIN, Morbidity Assessment Index for Newborns.

All 23 studies accounted for some form of inherent factor or diseases and adverse conditions of the newborn. The risk factors that were most widely studied and most consistently associated with LOS-NICU were the following: birth weight (60.9%,14/23) (9, 18, 24–27, 30, 32, 34, 36, 37, 40–42); gestational age (43.5%,10/23) (18, 24–26, 30–33, 40, 41); sepsis (30.4%,7/23) (9, 24, 30, 31, 33, 37, 41); necrotizing enterocolitis (NEC) (21.7%,5/23) (9, 24, 33, 36, 37); bronchopulmonary dysplasia (BPD) (21.7%,5/23) (9, 24, 33, 36, 37); and retinopathy of prematurity (ROP) (17.2%,4/23) (9, 30, 33, 36). Although most studies used congenital anomaly as an exclusion criterion, four studies suggested that congenital anomaly may be a risk factor for LOS-NICU (24, 28, 30, 37). Eight studies considered some form of antenatal care and maternal factors, and five concluded that the mode of delivery affected LOS-NICU (31–34, 41). Ten studies considered the effect of certain clinical scores and laboratory indicators on LOS-NICU, identified six relevant clinical scores, and three laboratory indicators. Five studies attempted to account for the treatment of the newborn (9, 24, 28, 31, 43). However, there was little consensus on what factors were appropriate. Five studies considered organizational factors (9, 27, 32–34), most of which were related to the setting of the care being received, including transfers between units.

## 4. Discussion

Our study was the first systematic review of risk factors for the LOS-NICU of newborns. Among the 23 studies that we included, birth weight, gestational age, sepsis, NEC, BPD, and ROP were the critical risk factors that were most widely studied and consistently associated with LOS-NICU. The results of our systematic review provide an up-to-date comprehensive summary of the latest evidence. They will inform the development of interventions to reduce LOS-NICU and prevent prolonged LOS-NICU of newborns.

Our results were consistent with previous findings that birth weight and gestational age are the most important risk factors affecting LOS-NICU (18, 24–26, 30, 32, 40, 41). Although another study found no significant association between LOS-NICU and gestational age (37), this study only assessed preterm infants with birth weight less than 1,500 g in Israel, which may not be applicable to other neonatal populations. Prevention of prolonged LOS due to gestational age and low-birth-weight starts with a healthy pregnancy. Preterm parturition is a syndrome that is triggered by multiple factors. Clinicians and researchers play a key role in improving biochemical knowledge on preterm delivery, identifying risk factors, and developing interventions that can address this complex syndrome (44). The birth weight and gestational age, as inherent factors, have the advantage of being measured directly and objectively at birth. Therefore, some studies have used birth weight and gestational age to predict LOS-NICU (45–47). However, Bender et al. (18) and Hintz et al. (9) found that predictions of LOS-NICU of newborns would not be very accurate if only these inherent factors were considered and not combining them with other factors. Bender et al. established a predictive model of LOS-NICU including birth weight, gestational age, and two disease severity tools as predictors. The result showed that the addition of the first-week disease severity improved the prediction accuracy of LOS-NICU compared with including only birth weight and gestational age. In addition, male infants generally have a longer NICU stay than female infants (27, 31, 32, 34), and the LOS-NICU of newborns varies in ethnicity (27, 34, 42). However, there are currently no studies to explain the causes of these phenomena.

Congenital anomalies were often used as one of the exclusion criteria in some studies, while four of the other studies that considered congenital anomalies showed that it had a significant effect on length of stay (24, 28, 30, 37). Some congenital anomalies are unlikely to have an impact on LOS-NICU, while some severe anomalies or those requiring surgery may have a significant impact on LOS-NICU, such as gastroschisis. However, none of the studies that we included gave a clear definition of congenital anomalies, and there was no accepted list. In the available studies, we were unable to identify which specific congenital anomalies were the risk factors for LOS-NICU. Therefore, we should consider this issue in our future studies.

There is a controversy about whether antenatal treatment and maternal factors are risk factors for LOS-NICU. Five studies found that newborns delivered by the cesarean section were weaker, sicker, and born earlier than those born vaginally, leading to a longer LOS-NICU (31–34, 41). Nevertheless, three studies showed that they were not associated with a longer LOS-NICU despite the proportion of cesarean sections being high (30, 36, 40). They argued that cesarean sections are the result of unsatisfactory fetal conditions but not an individual confounding factor for LOS-NICU. Early evidence suggests that antenatal corticosteroids confer benefits on fetal lung maturation, and they are widely recommended for women at risk of preterm birth (48), but there is no certainty about their effect on LOS-NICU (27, 31, 34). Moreover, several maternal factors associated with LOS-NICU were revealed in a multicenter study in China (33), including primigravida, maternal hypertension, and cesarean section; however, these impacts were minimal. Therefore, more research is needed to determine the association between antenatal treatment or maternal factors and the LOS-NICU of newborns.

Neonatal diseases and adverse conditions are not only important risk factors for LOS-NICU but are also a leading cause of neonatal death. Fifteen million premature infants are born every year, and more than one million of them die from diseases caused by the prematurity itself (49). Infection is one of the most common adverse events in hospitalized newborns, which poses a threat to all newborns (50–52). The most common infections affecting LOS-NICU in our study included sepsis, NEC, and pneumonia. These diseases may require prolonged symptomatic treatment, anti-infective therapy, antishock therapy, nutritional support, and surgical treatment and pose a high risk of complications, resulting in a longer treatment time and LOS. A study from Poland showed that the median LOS-NICU of all infected newborns was twice as long as that of uninfected newborns (25). At the same time, the occurrence of one or more clinical infections, as well as the site of infection, had a different kind of impact on LOS-NICU. Studies related to the incidence, risk factors, causative microorganisms, and impact on LOS of neonatal infections are necessary to increase and maintain awareness of the impact of infections, to help develop local and international diagnostic and treatment guidelines, to facilitate adequate and appropriate resource allocation, and to help design multicenter interventional studies. In addition, neonatal acute kidney injury (AKI), ROP, and BPD are also essential diseases affecting LOS-NICU. A multinational multicenter study found that the overall incidence of AKI was 29.9%, and newborns with AKI had an independent mortality rate four times higher than those without AKI, with a longer independent LOS-NICU (29), which is consistent with the result of Charlton et al. (35).

Among the scores developed specifically for neonatal medicine, six are now widely used to predict LOS-NICU, namely the Apgar score, the Score for Neonatal Acute Physiology (SNAPPE), the Morbidity Assessment Index for Newborns (MAIN), the Critical Risk Index for Babies (CRIB), the Transport risk index of physiologic stability (TRIPS), and the Hassan scale. In contrast, the Hassan scale is a composite outcome score for neonatal morbidity that assesses the number of morbidities diagnosed during the hospitalization period following delivery (53). Patil et al. (38) demonstrated the correlation between this scale and LOS-NICU in a study that included 7,037 neonates from 1997 to 2018 and concluded that the Hassan scale distinguished between long-stay and short-stay newborns better than other composite scores. In addition to neonatal scores, laboratory indicators during neonatal hospitalization also affected LOS-NICU. Ge et al. first explored the correlation between NT-proBNP7 levels and delayed discharge, showing that higher NT-proBNP7 levels were associated with a longer LOS-NICU (1). However, it is unclear whether heart function itself or disorders that affect heart function better explain this association.

Fewer studies have attempted to explain the effect of neonatal treatment factors and organizational factors on LOS-NICU. However, this is not easy, because even in the same country, different hospitals at the same level may provide different types of treatment and care to the newborn. Among limited studies on this topic, Zhang et al. compared LOS-NICU in independent children’s hospitals and perinatal prenatal centers having delivery facilities (33). Even after adjusting for neonatal morbidity, LOS-NICU remained significantly longer in children's hospitals than in prenatal centers, which may be related to the greater complexity of neonatal conditions at children’s hospitals and may also reflect differences in clinical care practices, healthcare policies, and availability of proper postdischarge medical care. Furthermore, two studies reached different conclusions about whether the transfer was a risk factor or not (9, 32), possibly emanating from different countries and different study populations.

This systematic review has the following limitations. First of all, while the search strategy was comprehensive and rigorous, it may still have missed including some studies. In addition, it is not feasible to perform a meta-analysis because of significant heterogeneity in the study design, study population, the definition of LOS-NICU, and statistical analysis methods. Finally, because most of the included studies were retrospective in nature, causal assertions could not be made regarding the risk factors for LOS-NICU.

In conclusion, we identified several of the most critical risk factors affecting LOS-NICU in published studies, including birth weight, gestational age, sepsis, NEC, BPD, and ROP. However, a few high-quality studies were available, and all included studies did not consider the effect of sociodemographic factors on LOS, such as family income, and parents’ psychological and health status. In addition to this, the level of NICU, home nursing, and antenatal and delivery room management may impact both severity of illness and LOS. Therefore, well-designed and more extensive prospective studies investigating the risk factors affecting LOS-NICU are still needed in the future. Applications of machine learning (ML) methods have been used extensively to solve various complex challenges in the field of medicine in recent years (54). ML methods are characterized by their ability to examine a lot of data and discover exciting relationships, provide interpretation, and identify patterns. ML can help enhance the reliability, performance, predictability, and accuracy of diagnostic systems for many diseases. Common machine learning classification models are Bayes network (BN) models, support vector machine (SVM), Radial basis function (RBF) tree, decision table, and naive Bayes. ML models have demonstrated high predictive performance in predicting LOS after pediatric heart transplantation and patients with COVID-19 (55, 56), which is a future direction for our research. In the meantime, studies should also focus on interventions to effectively reduce LOS-NICU and improve short- and long-term newborn outcomes.

## Data Availability

The original contributions presented in the study are included in the article/Supplementary Material, further inquiries can be directed to the corresponding author.
