# Supplementary material for: Risk factors for length of NICU stay of newborns: A systematic review
Source: Front Pediatr. 2023 Mar 13;11:1121406. doi: 10.3389/fped.2023.1121406 (PMC10040659; doi:10.3389/fped.2023.1121406)
Supplement: Supplementary file 1 [file Datasheet1.pdf]

Supplemental Digital : Database search strings

PubMed:

(((("Intensive Care Units, Neonatal"[Mesh]) OR ((((((((((("Newborn Intensive Care Unit"[Title/Abstract]) OR ("Neonatal Intensive Care Unit"[Title/Abstract])) OR ("Newborn Intensive Care Units"[Title/Abstract])) OR ("Neonatal ICU"[Title/Abstract])) OR ("Newborn ICU"[Title/Abstract])) OR ("ICU, Newborn"[Title/Abstract])) OR ("Newborn ICUs"[Title/Abstract])) OR ("Newborn Intensive Care Units"[Title/Abstract])) OR ("Neonatal Intensive Care Units"[Title/Abstract])) OR ("ICU, Neonatal"[Title/Abstract])) OR ("ICUs, Neonatal"[Title/Abstract])) OR ("Neonatal ICUs"[Title/Abstract])) OR (NICU[Title/Abstract]))) AND (("Infant, Newborn"[Mesh]) OR ((((((((((((("Infant, Newborn"[Title/Abstract]) OR ("Infants, Newborn"[Title/Abstract])) OR ("Newborn Infant"[Title/Abstract])) OR ("Newborn Infants"[Title/Abstract])) OR (Newborns[Title/Abstract])) OR (Newborn[Title/Abstract])) OR (Neonate[Title/Abstract])) OR (Neonates[Title/Abstract])) OR ("Infant, Low Birth Weight"[Title/Abstract])) OR ("Infant, Postmature"[Title/Abstract])) OR ("Infant, Premature"[Title/Abstract])) OR ("Infant, Small for Gestational Age"[Title/Abstract])) OR ("Infant, Very Low Birth Weight"[Title/Abstract])) OR ("Infant, Extremely Premature"[Title/Abstract])) OR ("preterm infants"[Title/Abstract])))) AND (("Length of Stay"[Mesh]) OR (((((((("length of stay"[Title/Abstract]) OR ("Stay Length"[Title/Abstract])) OR ("Stay Lengths"[Title/Abstract])) OR ("Hospital Stay"[Title/Abstract])) OR ("Hospital Stays"[Title/Abstract])) OR ("Stay, Hospital"[Title/Abstract])) OR ("Stays, Hospital"[Title/Abstract])) OR ("length of hospital stay"[Title/Abstract])) OR (LOS[Title/Abstract])))) AND (("Risk Factors"[Mesh]) OR ((((((((((((((("risk factors"[Title/Abstract]) OR (determinants[Title/Abstract])) OR ("clinical predictors"[Title/Abstract])) OR (predictors[Title/Abstract])) OR ("Factor, Risk"[Title/Abstract])) OR ("Risk Factor"[Title/Abstract])) OR ("influencing factors"[Title/Abstract])) OR ("influencing factor"[Title/Abstract])) OR (indicators[Title/Abstract])) OR (causality[Title/Abstract])) OR (cause[Title/Abstract])) OR (causes[Title/Abstract])) OR (factors[Title/Abstract])) OR (factor[Title/Abstract])) OR ("Risk Assessment"[Title/Abstract])) OR ("risk assessments"[Title/Abstract]))))

Web of science

#1 ((((((((((((((((((((((((TS=(“Intensive Care Units, Neonatal”)) OR TI=(“Newborn Intensive Care Unit”)) OR AB=(“Newborn Intensive Care Unit”)) OR TI=(“Neonatal Intensive Care Unit”)) OR AB=(“Neonatal Intensive Care Unit”)) OR TI=(“Newborn Intensive Care Units”)) OR AB=(“Newborn Intensive Care Units”)) OR TI=(“Neonatal ICU”)) OR AB=(“Neonatal ICU”)) OR TI=(“Newborn ICU”)) OR AB=(“Newborn ICU”)) OR TI=(“ICU, Newborn”)) OR AB=(“ICU, Newborn”)) OR TI=(“Newborn ICUs”)) OR AB=(“Newborn ICUs”)) OR TI=(“Newborn Intensive Care Units”)) OR AB=(“Newborn Intensive Care Units”)) OR TI=(“Neonatal Intensive Care Units”)) OR AB=(“Neonatal Intensive Care Units”)) OR TI=(“ICU, Neonatal”))

OR AB=(“ICU, Neonatal”)) OR TI=(“ICUs, Neonatal”)) OR AB=(“ICUs, Neonatal”)) OR TI=(“Neonatal ICUs”)) OR AB=(“Neonatal ICUs”)) OR TI=(NICU)) OR AB=(NICU)

#2 ((((((((((((((((((((((((((((TS=(“Infant, Newborn”)) OR TI=(“Infants, Newborn”)) OR AB=(“Infants, Newborn”)) OR TI=(“Newborn Infant”)) OR AB=(“Newborn Infant”)) OR TI=(“Newborn Infants”)) OR AB=(“Newborn Infants”)) OR TI=(Newborns)) OR AB=(Newborns)) OR TI=(Newborn)) OR AB=(Newborn)) OR TI=(Neonate)) OR AB=(Neonate)) OR TI=(Neonates)) OR AB=(Neonates)) OR TI=(“Infant, Low Birth Weight”)) OR AB=(“Infant, Low Birth Weight”)) OR TI=(“Infant, Postmature”)) OR AB=(“Infant, Postmature”)) OR TI=(“Infant, Premature”)) OR AB=(“Infant, Premature”)) OR TI=(“Infant, Small for Gestational Age”)) OR AB=(“Infant, Small for Gestational Age”)) OR TI=(“Infant, Very Low Birth Weight”)) OR AB=(“Infant, Very Low Birth Weight”)) OR TI=(“Infant, Extremely Premature”)) OR AB=(“Infant, Extremely Premature”)) OR TI=(“preterm infants”)) OR AB=(“preterm infants”)

#3 ((((((((((((((((TS=(“length of stay”)) OR TI=(“Stay Length”)) OR AB=(“Stay Length”)) OR TI=(“Stay Lengths”)) OR AB=(“Stay Lengths”)) OR TI=(“Hospital Stay”)) OR AB=(“Hospital Stay”)) OR TI=(“Hospital Stays”)) OR AB=(“Hospital Stays”)) OR TI=(“Stay, Hospital”)) OR AB=(“Stay, Hospital”)) OR TI=(“Stays, Hospital”)) OR AB=(“Stays, Hospital”)) OR TI=(LOS)) OR AB=(LOS)) OR TI=(“length of hospital stay”)) OR AB=(“length of hospital stay”)

#4 ((((((((((((((((((((((((((((((TS=(“risk factors”)) OR TI=(determinants)) OR AB=(determinants)) OR TI=(“clinical predictors”)) OR AB=(“clinical predictors”)) OR TI=(predictors)) OR AB=(predictors)) OR TI=(“Factor, Risk”)) OR AB=(“Factor, Risk”)) OR TI=(“Risk Factor”)) OR AB=(“Risk Factor”)) OR TI=(“influencing factors”)) OR AB=(“influencing factors”)) OR TI=(“influencing factor”)) OR AB=(“influencing factor”)) OR TI=(indicators)) OR AB=(indicators)) OR TI=(causality)) OR AB=(causality)) OR TI=(cause)) OR AB=(cause)) OR TI=(causes)) OR AB=(causes)) OR TI=(factors)) OR AB=(factors)) OR TI=(factor)) OR AB=(factor)) OR TI=(“Risk Assessment”)) OR AB=(“Risk Assessment”)) OR TI=(“risk assessments”)) OR AB=(“risk assessments”)

#5 #1 AND #2 AND #3 AND #4

Embase

#1'neonatal intensive care unit'/exp

#2'intensive care units, neonatal' OR 'newborn intensive care unit' OR 'neonatal intensive care unit' OR 'neonatal icu' OR 'newborn icu' OR 'icu, newborn' OR 'icus, newborn' OR 'newborn icus' OR 'newborn intensive care units' OR 'neonatal intensive care units' OR 'icu, neonatal' OR 'icus, neonatal' OR 'neonatal icus' OR nicu:ab,kw,ti

#3#1 OR #2

#4'newborn'/exp

#5'infant, newborn' OR 'infants, newborn' OR 'newborn infant' OR 'newborn infants' OR newborns OR newborn OR neonate OR neonates OR 'infant, low birth weight' OR 'infant, postmature' OR 'infant, premature' OR 'infant, small for gestational age' OR 'infant, very low birth weight' OR 'infant, extremely premature' OR 'preterm infants':ab,kw,ti

#6#4 OR #5

#7'length of stay'/exp

#8'stay length' OR 'stay lengths' OR 'hospital stay' OR 'hospital stays' OR 'stay, hospital' OR 'stays, hospital' OR los OR 'length of hospital stay':ab,kw,ti

#9#7 OR #8

#10'risk factor'/exp

#11'risk factors' OR determinants OR 'clinical predictors' OR predictors OR 'factor, risk' OR 'risk factor' OR 'influencing factors' OR 'influencing factor' OR indicators OR causality OR cause OR causes OR factors OR factor OR 'risk assessment' OR 'risk assessments':ab,kw,ti

#12#10 OR #11

#13#3 AND #6 AND #9 AND #12

Cochrane library

#1MeSH descriptor: [Intensive Care Units, Neonatal] explode all trees

#2("Newborn Intensive Care Unit" OR "Neonatal Intensive Care Unit" OR "Newborn Intensive Care Units" OR "Neonatal ICU" OR "Newborn ICU" OR "ICU, Newborn" OR "ICUs, Newborn" OR "Newborn ICUs" OR "Newborn Intensive Care Units" OR "Neonatal Intensive Care Units" OR "ICU, Neonatal" OR "ICUs, Neonatal" OR "Neonatal ICUs" OR NICU):ti,ab,kw

#3#1 OR #2

#4MeSH descriptor: [Infant, Newborn] explode all trees

#5("Infants, Newborn" OR "Newborn Infant" OR "Newborn Infants" OR Newborns OR Newborn OR Neonate OR Neonates OR "Infant, Low Birth Weight" OR "Infant, Postmature" OR "Infant, Premature" OR "Infant, Small for Gestational Age" OR "Infant, Very Low Birth Weight" OR "Infant, Extremely Premature" OR "preterm infants"):ti,ab,kw

#6#4 OR #5

#7MeSH descriptor: [Length of Stay] explode all trees

#8("Stay Length" OR "Stay Lengths" OR "Hospital Stay" OR "Hospital Stays" OR "Stay, Hospital" OR "Stays, Hospital" OR LOS OR "length of hospital stay"):ti,ab,kw

#9#7 OR #8

#10MeSH descriptor: [Risk Factors] explode all trees

#11(determinants OR “clinical predictors” OR predictors OR “Factor, Risk” OR “Risk Factor” OR “influencing factors” OR “influencing factor” OR indicators OR causality OR cause OR causes OR factors OR factor OR “Risk Assessment” OR “risk assessments”):ti,ab,kw

#12#10 OR #11

#13#3 AND #6 AND #9 AND #12
